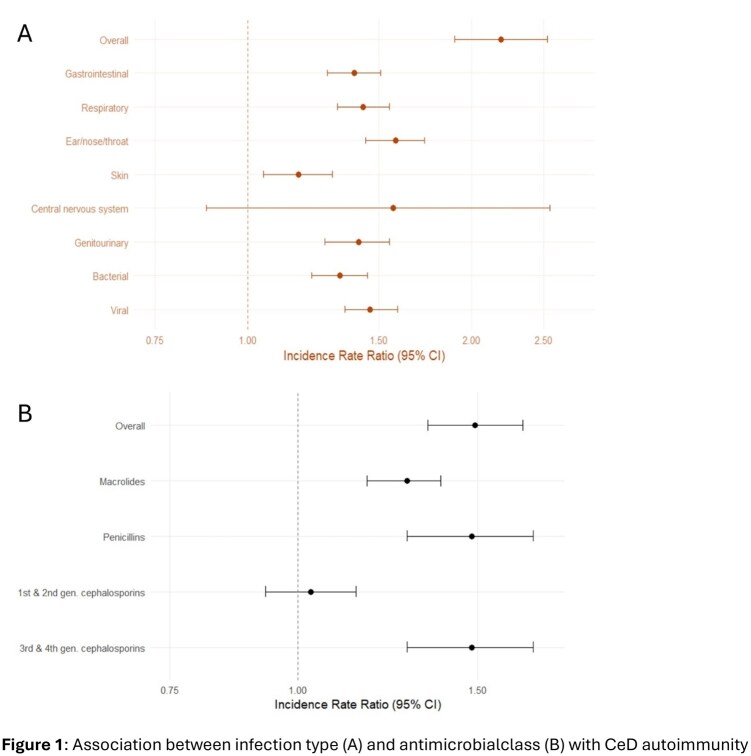# Poster Session I – Poster of Distinction I - A78 RISK OF CELIAC DISEASE AUTOIMMUNITY FROM EARLY-LIFE INFECTIONS AND ANTIMICROBIAL USE: A 10-YEAR RETROSPECTIVE BIRTH COHORT STUDY

**DOI:** 10.1093/jcag/gwaf042.078

**Published:** 2026-02-13

**Authors:** Q Goddard, J Bakal, H W Barkema, J K Holodinsky, G G Kaplan, B Li, D Nobrega, T Williamson, E Youngson, J A King

**Affiliations:** Community Health Sciences, University of Calgary, Calgary, AB, Canada; Alberta Health Services, Edmonton, AB, Canada; Community Health Sciences, University of Calgary, Calgary, AB, Canada; Community Health Sciences, University of Calgary, Calgary, AB, Canada; Community Health Sciences, University of Calgary, Calgary, AB, Canada; Alberta Health Services, Edmonton, AB, Canada; Community Health Sciences, University of Calgary, Calgary, AB, Canada; Community Health Sciences, University of Calgary, Calgary, AB, Canada; Alberta Health Services, Edmonton, AB, Canada; Community Health Sciences, University of Calgary, Calgary, AB, Canada

## Abstract

**Background:**

Previous studies report mixed findings regarding the connection between pediatric infection or antimicrobial use and the development of celiac disease (CeD). Accordingly, we investigate this association in further granularity.

**Aims:**

Estimate the risk of CeD autoimmunity from early life infections and/or antimicrobial use.

**Methods:**

We conducted a population-based cohort study including 464,203 live births in Alberta (2012–2021). Mothers were linked to antimicrobial dispensations during pregnancy. Newborns were linked to tissue transglutaminase (tTG) test results, antimicrobial dispensations, and infections indicated as the primary diagnosis in inpatient, emergency, and/or community-based physician visits. Poisson regression estimated incidence rate ratios (IRRs) of tTG-positivity based on exposure to infections/antimicrobials, adjusting for maternal age, maternal CeD status, and newborn sex. Conditional logistic regression estimated odds ratios (ORs) for developing CeD, matched within birthing parent. A mediation analysis was used to evaluate antimicrobial exposure as a potential mediator.

**Results:**

There were 2,520 cases of incident CeD autoimmunity in the cohort. No increased risk was associated with *in utero* exposure (IRR=1.00, 95% CI: 0.90–1.09). We observed an increased risk of CeD autoimmunity following early-life infection overall (IRR=2.19, 95% CI: 1.90–2.53) as well as across specific infection types (Figure 1A). Risk increased 7% for each additional infection (*p*<0.001). Antimicrobial exposure also increased risk of CeD autoimmunity (IRR=1.49, 95% CI: 1.34–1.66), with risk of CeD autoimmunity increasing 2% per additional antimicrobial dispensation (*p*<0.001). This association was present across multiple classes of antibiotics (Figure 1B). Similarly, complete lack of early-life exposure to antimicrobials was protective of CeD autoimmunity (IRR=0.68, 95% CI: 0.61–0.76), although infection was still associated with increased risk among those with no documented antimicrobial use (IRR=1.88, 95% CI: 1.52–2.33). We identified significant but modest mediation from antimicrobials (12.1%, 95% CI: 6.0–17.9%; *p*<0.001). While an association with infection remained when matching within birthing parent, it was less pronounced (OR = 1.33, 95% CI: 1.02–1.75) and the association with antimicrobial use disappeared (OR = 1.07, 95% CI: 0.85–1.34).

**Conclusions:**

Our findings support an association between incident CeD autoimmunity and both early childhood infection and antimicrobial use, with infection appearing to drive the association. However, the results from sibling-specific analyses suggests residual confounding could explain some of the relationship between exposures and CeD autoimmunity.

**Funding Agencies:**

Provincial Research Data Services, Alberta Health Services